# Improving Adherence to Physical Health Monitoring for Patients Prescribed Antipsychotic Medications Within a Perinatal Mental Health Community Team

**DOI:** 10.1192/bjo.2026.11441

**Published:** 2026-06-30

**Authors:** Nicola Gallagher, Bosky Nair, Anya Fearn, Joanne Pullen

**Affiliations:** 1 Maidstone & Tunbridge Wells NHS Trust, Maidstone, United Kingdom; 2 Kent and Medway Mental Health NHS Trust, Maidstone, United Kingdom

## Abstract

**Aims:**

Antipsychotic medications are commonly prescribed for psychotic illness, bipolar affective disorder and treatment-resistant depression. They are associated with significant physical health risks, including metabolic syndrome, cardiovascular complications, and extrapyramidal side effects. The National Institute for Health and Care Excellence (NICE) recommends comprehensive physical health monitoring at baseline, three months, and twelve months following initiation of antipsychotic treatment. Recommended physical health parameters include blood tests, body mass index (BMI) or body weight, blood pressure, heart rate, electrocardiogram (ECG) for certain antipsychotics, and side-effect profiles.

Antipsychotic medications can be safely prescribed to patients in the perinatal period, however, it is necessary to monitor their physical health. This project aimed to evaluate adherence to the aforementioned NICE guidelines, for all patients prescribed antipsychotic medications within a perinatal mental health community service (PMHCS).

**Methods:**

A retrospective review of all patients under the PMHCS caseload as of 1 August 2025 was conducted (n=388). Electronic case notes were reviewed to identify patients prescribed antipsychotic medication. Data collected included diagnosis, medication prescribed, duration of treatment and completion of recommended physical health monitoring in comparison to NICE guidance.

**Results:**

Of the 92 patients (24%) prescribed antipsychotic medication, 53 (58%) had medication initiated by PMHCS. The most common diagnosis was Bipolar Affective Disorder (34%) and quetiapine was the most commonly prescribed antipsychotic medication (51%).

Just over half of the cohort had baseline monitoring recorded (n=47, 51%) but certain parameters were commonly omitted; only 20% had Hbs-392c recorded and 34% had a blood pressure recorded. A total of 75 patients had been taking an antipsychotic medication for over three months, of which 31 (41%) had physical health monitoring to reflect this. There were 57 patients who had taken an antipsychotic medication for at least one year and amongst them, 31 (54%) had physical health monitoring.

**Conclusion:**

Our results demonstrated that despite NICE guidance outlining the required monitoring, it was uncommon for patients within this cohort to have the necessary investigations recorded at the required intervals. To address this, the team devised a structured antipsychotic monitoring strategy that included patient education, dissemination of patient information leaflets on physical health monitoring, distribution of monitoring equipment amongst staff, staff training, and recruitment of dedicated physical health nurses. Designated physical health clinic days and systematic data recording were planned to improve monitoring. A re-audit is planned to evaluate the impact of these interventions on compliance with physical health monitoring standards.

